# Reduced *LYNX1* expression in transcriptome of human iPSC-derived neural progenitors modeling fragile X syndrome

**DOI:** 10.3389/fcell.2022.1034679

**Published:** 2022-11-21

**Authors:** Karo Talvio, Rimante Minkeviciene, Kayla G. Townsley, Venkat Swaroop Achuta, Laura M. Huckins, Padraic Corcoran, Kristen J. Brennand, Maija L. Castrén

**Affiliations:** ^1^ Department of Physiology, Faculty of Medicine, University of Helsinki, Helsinki, Finland; ^2^ Pamela Sklar Division of Psychiatric Genomics, Department of Genetics and Genomics, Icahn Institute of Genomics and Multiscale Biology, Icahn School of Medicine at Mount Sinai, New York, NY, United States; ^3^ Nash Family Department of Neuroscience, Friedman Brain Institute, Icahn School of Medicine at Mount Sinai, New York, NY, United States; ^4^ Graduate School of Biomedical Science, Icahn School of Medicine at Mount Sinai, New York, NY, United States; ^5^ Department of Neurology, Medical University of Vienna, Vienna, Austria; ^6^ Division of Molecular Psychiatry, Department of Psychiatry, Yale University, New Haven, CT, United States; ^7^ Array and Analysis Facility, Department of Medical Sciences, Uppsala University, Uppsala, Sweden; ^8^ Department of Genetics, Yale University, New Haven, CT, United States

**Keywords:** fragile X syndrome, Epilepsy, LYNX1, neural progenitors, pluripotent stem cells, cholinergic signaling

## Abstract

Lack of FMR1 protein results in fragile X syndrome (FXS), which is the most common inherited intellectual disability syndrome and serves as an excellent model disease to study molecular mechanisms resulting in neuropsychiatric comorbidities. We compared the transcriptomes of human neural progenitors (NPCs) generated from patient-derived induced pluripotent stem cells (iPSCs) of three FXS and three control male donors. Altered expression of *RAD51C, PPIL3, GUCY1A2, MYD88, TRAPPC4, LYNX1,* and *GTF2A1L* in FXS NPCs suggested changes related to triplet repeat instability, RNA splicing, testes development, and pathways previously shown to be affected in FXS. LYNX1 is a cholinergic brake of tissue plasminogen activator (tPA)-dependent plasticity, and its reduced expression was consistent with augmented tPA-dependent radial glial process growth in NPCs derived from FXS iPSC lines. There was evidence of human iPSC line donor-dependent variation reflecting potentially phenotypic variation. NPCs derived from an FXS male with concomitant epilepsy expressed differently several epilepsy-related genes, including genes shown to cause the auditory epilepsy phenotype in the murine model of FXS. Functional enrichment analysis highlighted regulation of insulin-like growth factor pathway in NPCs modeling FXS with epilepsy. Our results demonstrated potential of human iPSCs in disease modeling for discovery and development of therapeutic interventions by showing early gene expression changes in FXS iPSC-derived NPCs consistent with the known pathophysiological changes in FXS and by revealing disturbed FXS progenitor growth linked to reduced expression of LYNX1, suggesting dysregulated cholinergic system.

## Introduction

Autism spectrum disorder (ASD), epilepsy, and varying degree of developmental delay commonly occur simultaneously (Tuchman and Cuccaro, 2011) and a number of overlapping genes are associated with these disorders (Zhu et al., 2014; Heyne et al., 2018; Ball et al., 2020; Leblond et al., 2021). A large locus heterogeneity of mutated genes even in clinically well-defined phenotypes complicates development of improved treatment strategies for the neurodevelopmental disorders (Woodbury-Smith et al., 2018). Furthermore, interaction of the genetic factors with epigenetics and environmental factors plays a role in the etiopathology of several cases increasing heterogeneity (Zahir and Brown, 2011). Since genes with diverse function can be functionally interconnected, pathway-based analyses offer insight into patient stratification and new treatment approaches. Approaches focusing on single-gene disorders with relatively uniform clinical manifestation such as fragile X syndrome (FXS) reduce the complexity of studying defective signaling pathways contributing to neurodevelopmental defects.

FXS is the most common inherited intellectual disability syndrome with an estimated prevalence of around 1 in 4,000 males and 1 in 8,000 females (Hagerman, 2008). Neurobehavioral phenotype in FXS varies from mild cognitive and learning defects to severe intellectual disability (Hagerman et al., 2012). Symptoms include hyperactivity and attention deficits, sensory integration problems, communication difficulties, poor motor coordination, social anxiety, and restricted repetitive and stereotyped patterns of behavior (Kaufmann et al., 2004; Clifford et al., 2007; Hagerman et al., 2010; Kaufmann et al., 2017). Approximately one-third of FXS males may be diagnosed with ASD (Hagerman et al., 2010; Richards et al., 2015), whereas roughly one in five suffers epileptic seizures (Louhivuori et al., 2009; Hagerman et al., 2012; Cowley et al., 2016), and a dual diagnosis of FXS and ASD increases the risk of epilepsy (Kaufmann et al., 2004).

FXS is caused by absence of the fragile X mental retardation protein (FMRP), most commonly due to a CGG trinucleotide repeat expansion in the 5′ untranslated region of the *FMR1* gene, whose promoter becomes silenced after ∼10–12.5 weeks (Willemsen et al., 2002) of gestation by interaction of *FMR1* mRNA with its associated trinucleotide repeat DNA (Colak et al., 2014). FMRP is an RNA-binding protein that is essential for normal synapse maturation and function (Davis and Broadie, 2017). Dendritic spine density is increased, and spine morphology is immature in multiple cortical regions of FXS individuals and *Fmr1* knockout (KO) mice (He and Portera-Cailliau, 2013). *Fmr1* KO mice recapitulate the main FXS phenotype and show susceptibility to audiogenic seizures (AGSs) caused by increased activity of glutamatergic neurons and decreased GABAergic transmission in the auditory pathways (Rotschafer and Razak, 2014; Rotschafer et al., 2015; Ethridge et al., 2017; Rotschafer and Cramer, 2017; Gonzalez et al., 2019). There is evidence that *Fmr1* deletion in VGLUT2-expressing neurons located in subcortical brain regions is sufficient to cause AGSs and that glutamatergic neurons in the inferior colliculus contribute to the AGS phenotype (Gonzalez et al., 2019).

Human induced pluripotent stem cells (iPSCs) can be used to model tissue differentiation and the onset of pathology in inherited disorders (Marchetto et al., 2011). Patient-specific iPSCs offer possibilities to study molecular mechanisms leading to the manifestation of comorbid symptoms. Previous studies have shown abnormalities in neuronal cells derived from iPSCs reprogrammed from somatic cells of FXS males (Bhattacharyya et al., 2008; Telias et al., 2015; Bhattacharyya and Zhao, 2016; Achuta et al., 2017; Achuta et al., 2018; Utami et al., 2020). Temporal gene expression changes of FXS neural progenitor cells (NPCs) can have long-lasting effects on brain development and influence lifelong neurogenesis in restricted areas of the adult brain (Telias et al., 2013; Das Sharma et al., 2020). We compared genome-wide gene expression of NPCs derived from three patient-specific FXS and control male iPSC lines using high-density oligonucleotide expression array. We found that FXS-specific gene expression changes in NPCs were consistent with the molecular and clinical FXS phenotype. Gene expression abnormalities in FXS NPCs associated with signaling pathways identified as potential treatment targets in FXS, highlighting the value of human iPSC models in development of treatment strategies. Furthermore, individual phenotype-related variation in molecular signaling might affect treatment responses and possibly contribute to failure of several clinical trials despite promising results in animal studies.

## Materials and methods

### Human neural progenitor differentiation in neurospheres

We used previously generated human iPSC lines from 3 FXS males (cell lines HEL100.1, HEL100.2, HEL69.5, and HEL70.3) and 3 control cell lines (HEL46.11, HEL23.3, and HEL11.4) reported in multiple previous studies (Achuta et al., 2017; Achuta et al., 2018; Danesi et al., 2018). The skin and blood samples were donated after informed consent and somatic cells were reprogrammed to human iPSC lines at Biomedicum Stem Cell Center (University of Helsinki, Finland) using Sendai virus (CytoTune-iPS Sendai Reprogramming Kit, Gibco, Life Technologies Ltd.). Multiple clones of iPSCs derived from each patient such as HEL100.1 and HEL100.2 (Achuta et al., 2018) were characterized. To increase privacy protection and security of the data associated with the human cell lines, the FXS iPSC lines have been anonymized. The donor information includes comorbid epilepsy. The present research was approved by the Ethical Committee of the Hospital District of Helsinki and Uusimaa.

Human pluripotent cells were sustained as monolayer cultures on Matrigel (BD Biosciences) coated plates in Essential 8 (E8) medium with E8 supplement (both from Gibco) in a humidified incubator. The culture medium was changed every other day and colonies were passaged every 4–5 days using 0.5 mM ethylenediaminetetraacetic acid (EDTA; Invitrogen, Life Technologies Ltd.) in phosphate-buffered saline (PBS). Temperature was maintained at 37°C and CO_2_ at 5%. *Mycoplasma* detection kit (MycoAlertTM, Lonza group Ltd.) was used to ensure cultures were free of *mycoplasma*.

Neuronal differentiation and culturing of neurospheres were performed as described previously (Nat et al., 2007; Achuta et al., 2017). Neurosphere formation from iPSC clusters was induced on low adherent plates in neuronal differentiation medium (NDM) containing Dulbecco’s Modified Eagle Medium (DMEM)/F-12, Neurobasal (1:1), 1 x B27 supplement, 2 mM Glutamax, 1 x N2 supplement (all from Gibco, Life Technologies Ltd.), and 20 ng/ml basic fibroblast growth factor (bFGF, PeproTech). On the first day, 10 μM of Y-27632 dihydrochloride (Abcam) was added to enhance neuronal differentiation. Free-floating aggregates referred to as embryoid bodies (EBs) were formed in 2–3 days. Medium was replaced every 2–3 days after the first week and EBs were passaged approximately once a week. After culturing for 6 weeks, cells were transformed to neural progenitor cells (NPCs), which grew in free-floating neurospheres. Neurosphere NPC (NS) samples were collected. For day 1 (D1) and day 7 (D7) NPC samples, 15–20 neurospheres (sized on average ∼200–250 µm) were placed on poly-D-lysine/laminin (Sigma-Aldrich) coated cover glasses and differentiated for 1 and 7 days respectively in NDM without bFGF, allowing early differentiation towards neuronal lineages to progress.

### Bulk RNA sequencing of human induced pluripotent stem cell-derived neuronal cells

NPCs, glutamatergic cells (iGLUTs), and GABAergic cells (iGABAs) were generated from human iPSCs lines derived from human peripheral blood mononuclear cells (PBMCs) of nine control donors without a history of neurodevelopmental, neurodegenerative, or neuropsychiatric disorders (3 XY, 6 XX) and maintained as described previously (Dobrindt et al., 2020). The cell lines are publicly available through the California Institute for Regenerative Medicine (CIRM) and have been fully characterized previously (Dobrindt et al., 2020). RNA was isolated from NPCs, DIV21 iGLUTs, and DIV35 iGABAs and RNA-seq libraries were prepared and sequenced by the New York Genome Center. The paired-end sequencing reads (125 bp) were generated on an Illumina HiSeq 2500 platform (coverage per reads: 40 M), aligned to hg38 using Rsubread (Liao, Smyth, Shi, 2019; uniquely mapping reads were counted with featureCount). Feature counts were transformed into counts per million (cpm), filtered, and log2 transformed.

### Immunocytochemistry

For immunocytochemistry of NPCs, cells were fixed with 4% paraformaldehyde in PBS for 10 min at room temperature (RT). After blocking nonspecific staining in PBS containing 10% normal goat serum (NGS), 1% bovine serum albumin (BSA) and 0.1% Triton X-100 for 45 min at RT, cells were incubated with the primary antibodies overnight at 4°C. Primary antibodies were diluted in PBS containing 1% NGS, 1% BSA and 0.1% Triton X-100. All primary antibodies used in the study are commercially available and well characterized. We used primary antibodies recognizing SOX2 (1:25, MAB 2018; R&D Systems), Nestin (1:200, SC-20978; Santa Cruz Biotechnologies), DCX (1:500, AB2253; Millipore), and MAP2 (1:2500, M4403; Sigma). Secondary antibodies were applied in PBS containing 1% BSA for 45 min at RT. Secondary antibodies were goat anti-chicken Alexa Fluor 488 (1:500, A-1139, Invitrogen), anti-rabbit Alexa Fluor 546 (1:500, A11003; Invitrogen), and anti-mouse Alexa Fluor 635 (1:500, A-31574; Invitrogen). After final washes, the cell nuclei were counterstained with Vectashield mounting media containing DAPI (Vector Laboratories).

### Microarray analysis

Transciptome analysis of NS, D1 and D7 NPCs samples was performed using Affymetrix Clariom D Human Array (Thermo Fisher Scientific). Total RNA (100 ng) was processed with a GeneChip WT PLUS reagent kit for the Affymetrix Array (Thermo Fisher Scientific) according to the sample preparation guide. Raw expression data were normalized using Expression Console program (v.1.3) (https://www.thermofisher.com/). The signal space transformation robust multi-array average (SST-RMA) method was used to normalize the data (Li and Wong, 2001; Irizarry et al., 2003). To identify differentially expressed genes, an empirical Bayes moderated *t*-test was applied using the “limma” package (version 3.34.9) (Smyth, 2004; Smyth et al., 2005). To address the problem of multiple testing, *p*-values were adjusted using the method of Benjamini and Hochberg (Benjamini and Hochberg, 1995). We report loci with adjusted *p*-values less than 0.05 as considered significantly differentially expressed. Changes in expression are shown as log2 (fold change), abbreviated as log2FC. The plotMD function of the limma package was used to illustrate correlations between the changes and average expression levels.

### Imaging of neural progenitor morphology with response to tissue plasminogen activator antibody

Morphologies of differentiating NPCs derived from the FXS cell lines HEL70.3 and HEL100.1 and from the control cell line HEL11.4 were monitored with time-lapse imaging for a period of 24 h (Supplementary Videos) in a cell-culturing instrument combined with a phase contrast microscopy, automation, and environmental control (Cell-IQ system; Chip-Man Technologies, Ltd., Tampere, Finland). The instrument consisted of an integrated incubator (±0.2 C), two incubation gas flow controllers, precision movement stages (*x*, *y* axes: ± 1 μm; *z* axis: ± 0.4 μm) and an automated optics module fully controlled by machine vision-based software and analysis software. The imaging system enabled continuous monitoring of adherent cells in two plates in an integrated plate holder. Neurospheres (15–20 neurospheres/well) were plated on 6-well plates in NDM without mitogens that allowed cell attachment to the surface and differentiation of progenitors. Polyclonal anti-human tissue plasminogen activator antibody (tPA ab #387; American Diagnostica) was added (200 ng/ml) to triplicate samples after plating. Imaging began 2.5 h after plating and machine vision enabled analysis of a continuous time-lapse image series of living cells for observing morphological changes during progenitor differentiation without the use of labels or dyes (Achuta et al., 2014; Achuta et al., 2017).

Cellular processes growing from differentiating NPCs of each cell line were measured with the ImageJ freeware. To analyze effect of tPA ab, the longest processes emanating from neurospheres were measured at 12 h and then at 6 h to determine growth. We analyzed the sizes of those neurospheres that were fully captured at the beginning of recording (2.5 h after plating) and at 6 h and 12 h to correlate for process growth.

### Intracellular Ca^2+^ imaging

The functional properties of NCPs were assessed using ratiometric intracellular Ca^2+^ ([Ca^2+^]_i_) recordings, which were performed as described previously (Achuta et al., 2017). For experiments, 15–20 neurospheres were plated on poly-D-lysine/laminin coated cover slips. Cells were differentiated for 7 days and incubated with 4 µM fura-2 acetoxymethylester (fura-2/AM, dissolved in dimethyl sulfoxide, Sigma) for 20 min at +37°C in HEPES-buffered medium (HBM, pH 7.4) consisting of 137 mM NaCl, 5 mM KCl, 0.44 mM KH_2_PO_4_, 4.2 mM NaHCO_3_, 2 mM CaCl_2_, 0.5 mM MgCl_2_, 10 mM glucose, and 1 mM probenicid. Cover slips were placed in a temperated perfusion chamber and perfused at 2 ml/min at +37°C. Cells were exposed to (S)-3,5-dihydroxyphenylglycine (DHPG, 10 μM; Abcam Biochemicals) and the fluorescence intensity was recorded using 340 nm and 380 nm light excitation through a filter changer under the control of the InCytIM-2 System (Intracellular Imaging) and a dichroic mirror (DM430, Nikon). Light emission was measured through a 510 nm barrier filter with an integrating charge-coupled device camera (COHU). An image (ratio 340 nm/380 nm) was acquired every second. Up to 100 cells derived from each neurosphere were recorded simultaneously. The data were analyzed with the InCyt 4.5 software and further processed with Origin 6.0 software (OriginLabCorp.).

### Statistical analysis

The Student’s *t*-test was used to assess differences of cellular process length. Amplitudes of [Ca^2+^]_i_ responses were compared with two-way ANOVA. Data are expressed as mean ± SEM. Unadjusted *p*-values < 0.05 were considered significant.

## Results

### Comparison of gene expression in fragile X syndrome and control human induced pluripotent stem cell-derived neural progenitors

**FIGURE 1:**
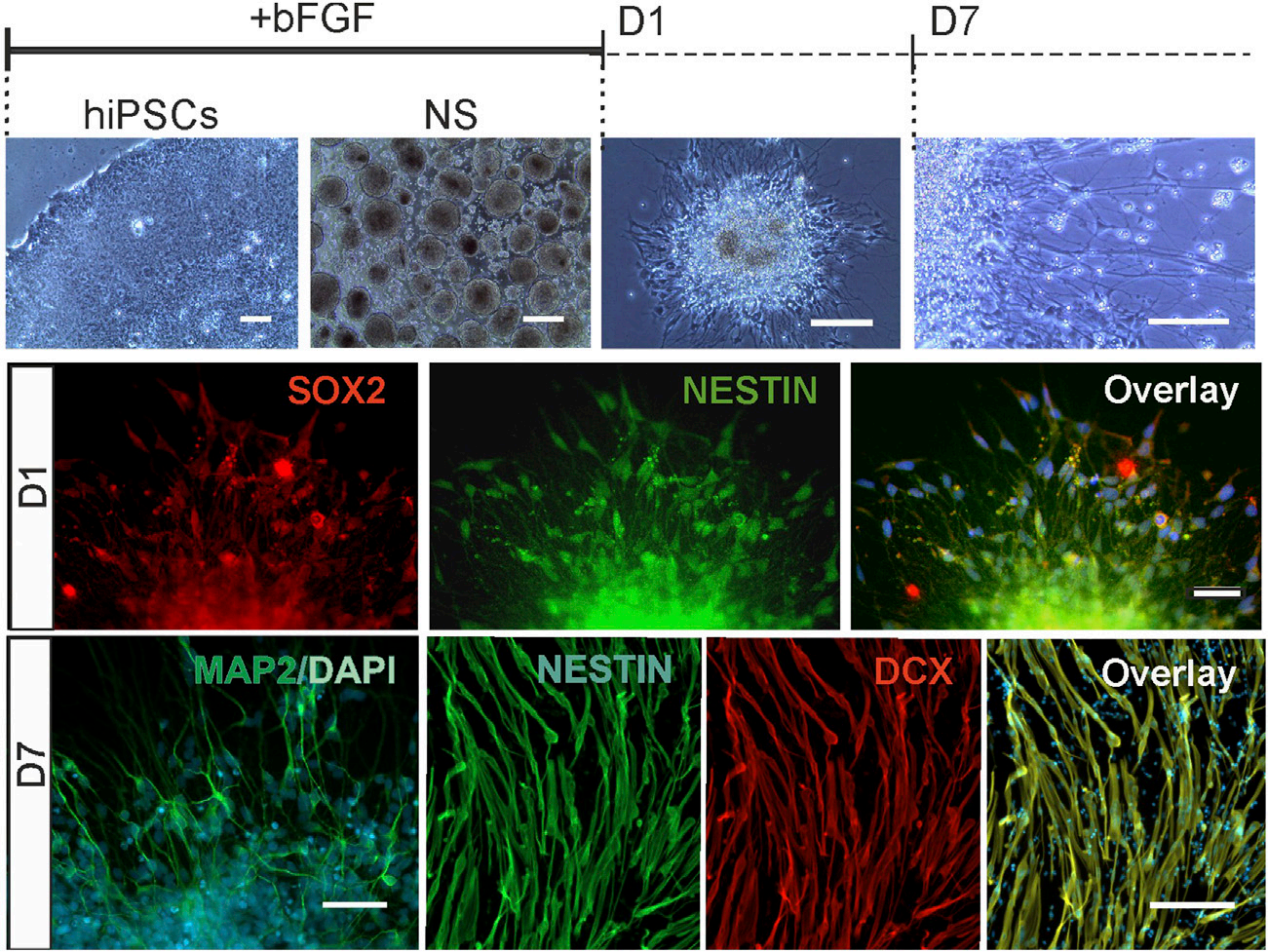
NPCs in human neurosphere model. Human iPSCs were differentiated to NPCs in neurospheres in the presence of mitogen (bFGF) and in neurospheres differentiated for 1 day (D1) and 7 days (D7) after withdrawal of mitogen. Representative bright field images of human iPSCs, free floading neurospheres, a differentiating neurosphere at D1, and cells migrated from the neurosphere at D7. All NPCs expressed pluripotency markers; **D1**: Expression of SOX2 (red), NESTIN (green) and their overlay with nuclear stain DAPI, 4′,6-diamidino-2-phenylindole (blue) in D1 NPCs. **D7**: Immunoreactive cells for neuronal marker MAP2 in a D7 neurophere, and cells immunostained with Doublecortin (DCX, red), Nestin (green) and their overlay with DAPI (blue). Scale bar 50 μm.

To identify FXS-specific changes in human NPCs lacking FMRP, we compared the genome-wide gene expression of NPCs derived from three patient-specific FXS and three control male iPSC lines using high density oligonucleotide expression array. Transcriptomes were analyzed in NPCs at three time points; 1) in proliferating neurospheres (NS), 2) at day 1 of differentiation (D1), and 3) at day 7 of differentiation (D7). NPCs grown in neurospheres were immature neuronal cells and expressed pluripotent markers at all studied time points (Figure 1). D1 and D7 NPCs were cultured without mitogens and progenitor early stage differentiation was seen as cell migration out from the neurosphere cell cluster and morphological transformation of cells into neuronal-like cells with expression of neuronal markers Doublecortin (DCX) and MAP2 along with pluripotency markers without formation of synaptic contacts (Figure 1). Previous studies have shown that cells migrated out from D1 and D7 human neurospheres display intracellular calcium responses to membrane depolarization with increased extracellular potassium (Danesi et al., 2018) and around 30% of cells respond to activation of glutamate receptors (Achuta et al., 2017; Achuta et al., 2018). Radial glia express type I metabotropic glutamate receptors and a small population of faster migrating cells with neuronal morphology respond to activation of ionotropic glutamate receptors in differentiating neurospheres. A cell population of less than 10% of cells respond to NMDA (Achuta et al., 2017). Differentiation towards glutamate responsive cells is increased in FXS NPCs (Achuta et al., 2018), but molecular mechanisms causing augmented fate determination towards glutamatergic lineages are not known.

**FIGURE 2:**
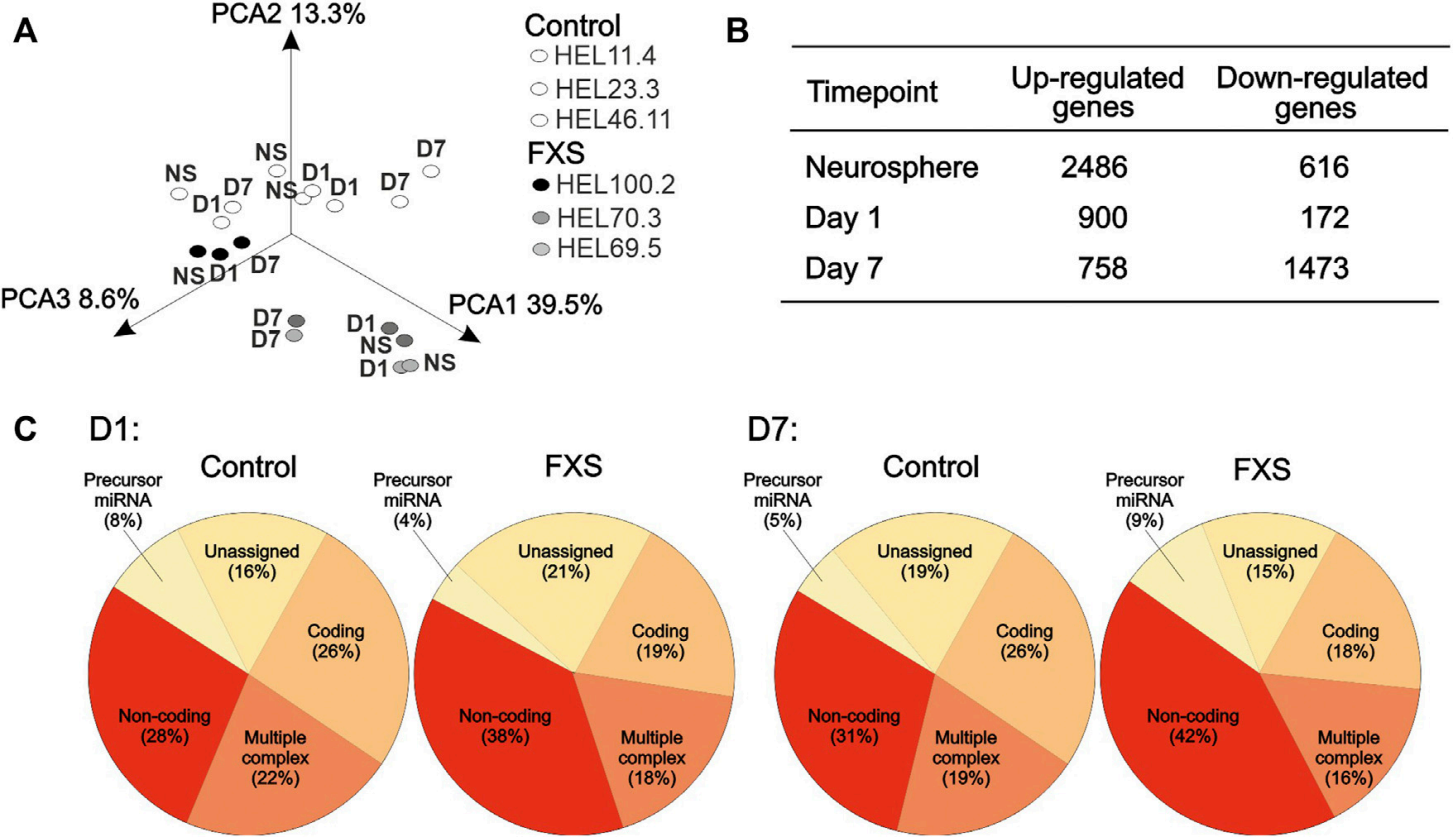
Exploratory analysis of transcriptome data. **(A)** Principal component analysis displays segregation of neurosphere (NS), day 1 (D1), and day 7 (D7) NPCs derived from FXS (HEL69.5, HEL70.3, and HEL100.2) and control (HEL46.11, HEL23.3, and HEL11.4) human iPSC lines. **(B)** Number of differently expressed transcripts (log2 fold change < −1 or >1 and unadjusted *p*-value < 0.05) identified between FXS and control NPCs before adjustment for multiple comparisons at each time point. **(C)** Relative proportion of differentially expressed RNAs by function in FXS and control human iPSC-derived NPCs after 1 day (D1) and 7 days (D7) of differentiation.

After data normalization, expression signal distributions were similar across all cell lines and time points studied. Principal component analysis (PCA) showed segregation of FXS and control samples in Figure 2A. An exploratory analysis of differential gene expression between FXS and control NPCs using less conservative filters (log2 fold change < −1 or >1 and unadjusted *p*-value < 0.05) gave a large number of genes (Figure 2B). Patterns of differential gene expression based on the locus type indicated that the expression of non-coding RNAs was increased in FXS NPCs compared to controls, while the expression of miRNAs was induced in FXS progenitors between D1 and D7 (Figure 2C) in agreement with the proposed role of FMRP in the regulation of miRNA maturation (Wan et al., 2017). Decreased mRNA complexity in FXS progenitors at D7 likely reflected reduced usage of alternative RNA isoforms.

The differential gene expression analysis performed using limma allowed us to refine the analysis and to reduce the number of differentially expressed genes. Applying the conservative filters and only considering loci with adjusted significances less than 0.05, *FMR1* was the only differently expressed gene both in NS and D1 NPCs (log2FCs −8.67 and −8.34, respectively, both *p* < 0.0001). *FMR1* was also the most significantly differentially expressed gene at D7. At D7, there were 11 differently regulated transcripts, including an unprofiled locus identified with the probe TC0700011304. hg.1 (log2FC 1.10, *p* = 0.023). The differently expressed 10 genes are shown in Table 1.

**TABLE 1:** Differently expressed genes between human iPSC-derived FXS and control D7 NPCs. *p*-values have been adjusted by the method of Benjamini and Hochberg (Benjamini and Hochberg, 1995).

Gene or probe	log_2_FC	*p*-value
*FMR1*	−8.84	0.000000085
*GTF2A1L*	−3.59	0.0040
*PPIL3*	2.24	0.0095
*RAD51C*	2.73	0.014
*GUCY1A2*	1.86	0.022
*MYD88*	2.42	0.033
*bochawby*	−1.72	0.033
*LYNX1*	−1.53	0.039
*RP11-485F13.1*	−1.74	0.039
*TRAPPC4*	1.42	0.044

Expression of *LYNX1* was reduced in FXS NPCs at D7, consistent with the decreased expression of *LYNX1* (log2FC = −4.36, *p* = 0.020) in human embryonic stem cell (ESC)-derived NPCs modeling FXS at D12 of differentiation in a previously reported on RNA sequencing analysis (Peteri et al., 2021). *LYNX1* binds to nicotinic acetylcholine receptors (nAChRs) and functions as an allosteric modulator, balancing neuronal activity and survival in the central nervous system (CNS) (Miwa et al., 1999; Ibanez-Tallon et al., 2002; Miwa et al., 2006; Miwa et al., 2011; Picciotto et al., 2012; Falk et al., 2021). Disturbed cholinergic signaling in FXS NPCs was supported by increased expression of *MYD88* and *TRAPPC4*. The inflammatory complex associated *MYD88* is a target for nAChRs (Li et al., 2011), and *TRAPPC4* encodes synbindin that is an organizer of cholinergic synapses (Zhou et al., 2021).

FXS NPCs showed the largest increase in the expression of *RAD51C*, which is a member of the RAD51 family, whose members are involved in the homologous recombination and repair of DNA. In addition, *PPIL3* and *GUCY1A2* displayed increased expression. *PPIL3* encodes peptidylprolyl isomerase-like 3 (Rajiv and Davis, 2018), a cyclophilin that functions as a spliceophilin regulating mRNA splicing. *GUCY1A2* codes for the alpha subunit of guanylate cyclase complex that catalyzes the conversion of GTP to 3′,5′-cyclic GMP (cGMP) and pyrophosphate (Koesling et al., 1991). Decreased cAMP and cGMP levels and increased activity of phosphodiesterase 2 (Pde2a), an enzyme that hydrolyzes cAMP and cGMP, are found in *Fmr1* KO mouse brain (Maurin et al., 2019).

Finally, transcripts for the testis specific transcription factor *GTF2A1L* (Upadhyaya et al., 1999), and the long-intervening/intergenic noncoding RNA (lincRNA) *RP11-485F13.1*, which are implicated in gonocyte differentiation (Winge et al., 2018), were decreased in FXS NPCs. Dysregulation of these gonocyte-related genes is potentially linked to abnormal testes development and macro-orchidism in FXS males (Lachiewicz and Dawson, 1994; Charalsawadi et al., 2017).

### Blocking tissue plasminogen activator prevents process growth in fragile X syndrome progenitors

Effects of LYNX1 on brain plasticity have been shown to be tPA dependent (Bukhari et al., 2015). We previously showed increased tPA expression in mouse NPCs lacking FMRP and in the brain of *Fmr1* KO mice. Blocking tPA function affected differentiation of early *Fmr1* KO mouse progenitors (Achuta et al., 2014). Progenitor populations differ between murine and human models, raising the question of whether tPA-mediated effects also exist in human FXS progenitors.

**FIGURE 3:**
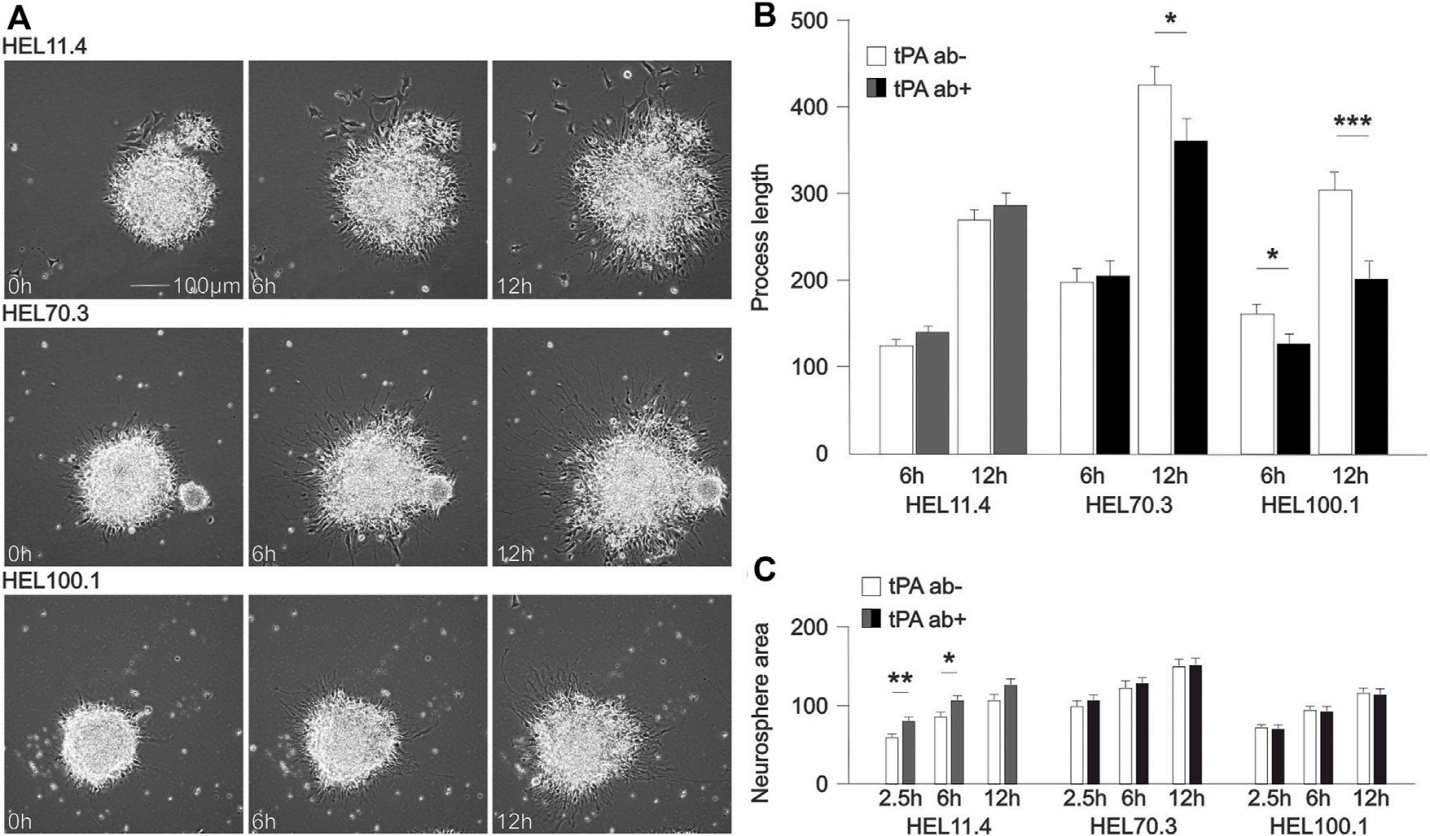
Tissue plasminogen activator (tPA)-mediated effects downstream of LYNX1 on cellular process growth. **(A)** Representative time-lapse images of neurospheres derived from control **(**HEL11.4) and FXS (HEL70.3 and HEL100.1) iPSC lines. Video clips of representative untreated and treated neurospheres derived from each cell line are shown in Supplementary Materials. **(B)** Distance extended by the furthest reaching cellular process emanating from control (HEL11.4) and FXS (HEL70.3 and HEL100.1) neurospheres 6 h and 12 h after plating on matrix in medium without mitogens allowing progenitor differentiation. The number of analyzed neurospheres in separate cultures (n) without/with tPA ab treatment: HEL11.4: 59 (*n* = 5)/70 (*n* = 5); HEL70.3: 34 (*n* = 3)/29 (n = 3); HEL100.1: 67 (n = 9)/29 (n = 4). **(C)** Neurosphere core size in control (HEL11.4) and FXS (HEL70.3 and HEL100.1) neurospheres 2.5 h (at the initiation of recording), 6 h, and 12 h after plating without and with tPA ab treatment. Human tPA ab (200 ng/ml) was added at the beginning of the differentiation as indicated (dark gray in control; black in FXS). Results are shown as mean ± SEM. Significances were determined with the Student’s *t*-test. *p < 0.05, **p < 0.01, ***p < 0.001.

We examined the effects of blocking tPA function with a neutralizing antibody on differentiating human FXS and control NPCs using time-lapse imaging during the first day of neurosphere differentiation (Figure 3A). Images of neurospheres were captured up to 24 h after plating (Supplementary Video). During the first hours, an extension of processes from the sphere was visible. We compared the extension of cell processes at 6 and 12 h time points in FXS and control spheres and found an effect of tPA ab after 12 h differentiation in FXS cells but not in control spheres (Figure 3B). To assess the potential effects of sphere size on process growth, we analyzed spheres that were fully imaged across all conditions (total of 197 spheres and processes), and found out that there was no correlation between sphere size and change in maximal process extension between 6 and 12 h of differentiation (Pearson correlation coefficient 0.183). However, the size of neurospheres showed variation between cell lines at the 2.5 h time point when time-lapse recording and analysis started (Figure 3C), likely reflecting increased proliferation of FXS progenitors and altered fate determination shown previously (Tervonen et al., 2009; Castrén, 2016; Utami et al., 2020). Exposure to tPA ab affected (initially similarly sized) neurosphere core growth in control, but this early change was not observed in FXS neurospheres (Figure 3C). The results supported disturbed tPA-regulated processes during early differentiation of FXS NPCs, likely associated with LYNX1 signaling.

### Expression of genes involved in *LYNX1*-related cholinergic signaling in fragile X syndrome neural progenitors

**FIGURE 4:**
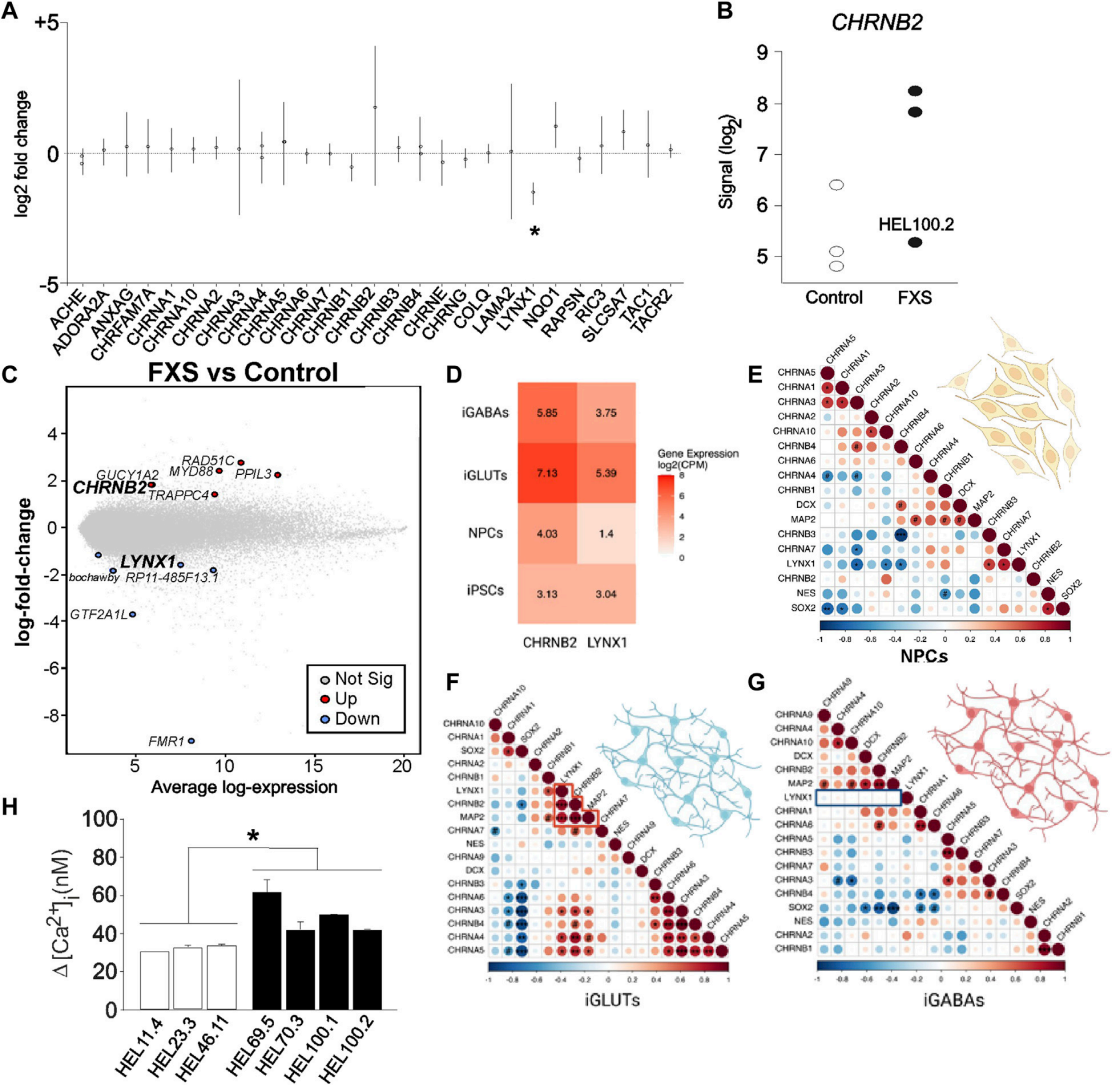
*LYNX1*-related gene expression in FXS NPCs. **(A)** The log2 fold expression changes of genes assigned Gene Ontology (GO) term GO:0007271; synaptic transmission, cholinergic in transcriptomes of D7 FXS NPCs compared with controls. Statistical significance in adjusted *p*-values. **(B)** Expression of *CHRNB2* in D7 FXS and control NPCs. **(C)** Gene expression changes in D7 FXS *versus* control NPCs plotted with the average expression levels of genes targeted in the array. The location of the significantly affected genes (adjusted *p*-value < 0.05) and the *CHRNB2* gene are highlighted to show their relatively high average expression values (*LYNX1* 7.496 and *CHRNB2* 6.328). **(D)** Average gene expression [log2(CPM+1)] of *CHRNB2* and *LYNX1* across iPSCs and neuronal cell-types in matched control donor lines (*n* = 9–11). **(E)** Gene expression of *LYNX1* correlated with different nAChR subtypes in NPCs. **(F)** *LYNX1* expression correlated with *CHRNB2* expression in human iPSC-derived glutamatergic neurons (iGLUTs), **(G)** but not in human iPSC-derived GABAergic neurons (iGABAs), suggesting a potential role in co-expression of LYNX1 and nAChR subtypes in fate determination of neuronal lineages. Created with BioRender.com. **(H)** The mean amplitude of [Ca^2+^]_i_ responses to type I mGluR agonist DHPG in D7 NPCs derived from control (HEL46.11, HEL23.3, and HEL11.4) and FXS (HEL69.5, HEL70.3, HEL100.1 and HEL100.2) iPSC lines. Results are shown as mean ± SEM. *p < 0.05.

Reduced *LYNX1* expression in FXS iPSC- and ESC-derived NPCs suggested dysregulated cholinergic signaling and motivated further comparison of gene expression in D7 NPCs derived from the three FXS (HEL69.5, HEL70.3, and HEL100.2) and the three control (HEL11.4, HEL23.3, and HEL46.11) iPSC lines with respect to putative changes in signaling pathways related to *LYNX1*. No specific pathway for LYNX1 was available and we explored gene expression changes of genes assigned Gene Ontology (GO) term GO:0007271, synaptic transmission, cholinergic, using the gconvert function in gprofiler2 (Figure 4A). We observed a noticeable high variation in the regulation of *CHRNB2* expression in FXS NPCs compared with controls, without reaching the level of significance (Figure 4A). *CHRNB2* expression was higher in HEL69.5 and HEL70.3 FXS NPCs than in HEL100.2 FXS NPCs (Figure 4B). *CHRNB2* encodes the *ß* subunit of nAChR (Nichols et al., 2014). Both *LYNX1* and *CHRNB2* were expressed in the tertile of high average expression of genes targeted in the array of D7 NPCs (Figure 4C).

LYNX1 can affect pentamer stoichiometry of nAChRs and may together with differential expression of nAChR subunits modulate cholinergic signaling and NPC fate determination. The *ß* subunit is essential in the nAChR α4β2 pentamer, which together with α7 nAChRs mediates modulatory activity of LYNX1, leading to sensitized effects of nicotine in the *Lynx1* KO mice (Nichols et al., 2014). RNA sequencing revealed *LYNX1* and *CHRNB2* expression across human iPSC-derived neural progenitors, glutamatergic neurons and GABAergic neurons (Figure 4D). Gene expression of LYNX1 was significantly correlated with different nAChR subtypes in NPCs expressing pluripotency markers (Figure 4E) and in iPSC-derived glutamatergic neurons (iGLUTs; Figure 4F) but was not correlated with *CHRNB2* gene expression in hiPSC-derived GABAergic neurons (iGABAs; (Figure 4G), suggesting a potential role for co-expression of LYNX1 and nAChR subtypes in fate determination of neuronal lineages. The data are consistent with *CHRNB2* expression by neural progenitors for GABAergic neurons and post-mitotic glutamatergic neurons reported previously (Alzu’bi et al., 2020).

### Altered expression of genes related to epilepsy in neural progenitors derived from the fragile X syndrome donor with clinical epilepsy

**FIGURE 5:**
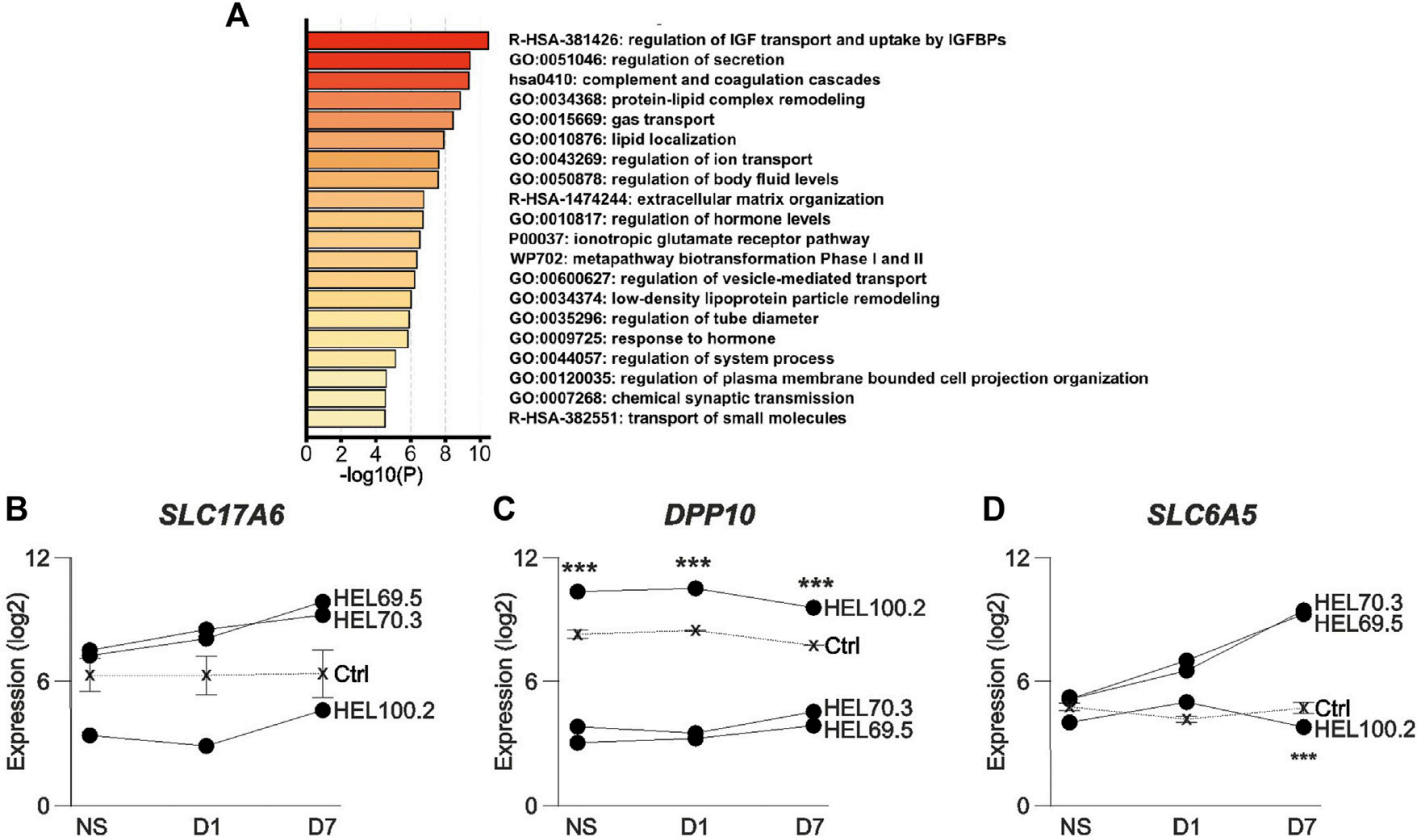
Pathway analysis and expression of genes associated with the mouse audiogenic seizure phenotype in human FXS NPCs generated from the donor with epilepsy and from donors without epilepsy. **(A)** Pathway gene enrichment analysis of differently expressed genes in HEL100.2 and other FXS NPCs in D7 NPCs. **(B)** *SLC17A6,* **(C)** *DPP10*, and **(D)** *SLC6A5* expression in neurosphere (NS), Day 1 (D1), and Day 7 (D7) NPCs derived from the FXS male donor with epilepsy (HEL100.2) and from FXS male donors without epilepsy (HEL70.3 and 69.5). Expression levels of three control iPSC-derived NPCs are shown with a dashed line. Data are shown as means ± SEM. *p*-values were adjusted with the method of Benjamini and Hochberg (Benjamini and Hochberg, 1995). ***p < 0.001 when comparing HEL100.2 and other FXS (HEL70.3 and 69.5) NPCs.

Our previous functional studies showed increased [Ca^2+^]_i_ responses to metabotropic glutamate receptor (mGluR) activation in human D7 in FXS NPCs compared to controls (Achuta et al., 2017). Amplitude of responses to the group I mGluR agonist DHPG was increased in all FXS NPCs and the responses of NPCs derived from different FXS iPSC lines did not differ (Figure 4). The donor of HEL100 cell lines was a FXS male with comorbid epilepsy and the question raised whether differences in transcriptomics of HEL100.2 D7 NPCs could reflect the epilepsy phenotype of the donor. In PCA, HEL100.2 derived NPCs segregated from the other FXS NPCs at all time points (Figure 2A). In the transcriptome of HEL100.2 NS, 397 loci were upregulated and 1204 downregulated when compared with transcriptome of the other FXS NS (adjusted *p* < 0.05). The differences diminished during culturing; at D7 67 loci were upregulated and 137 loci downregulated in HEL100.2 NPCs. Altogether 30 genes were dysregulated across all studied time points (Supplementary Table). Seven most significantly differently expressed genes at each time point are shown in Table 2. Many of these genes are associated with epilepsy and ictogenesis, including *DPP10*, *DOCK8*, *IAH1*, *LRRC4C,* and *SLC6A5* (Singh et al., 2006; Xiao et al., 2007; Carta et al., 2012; Wang et al., 2017; Parano et al., 2021). Metascape analysis with D7 data revealed that differentially expressed genes were particularly enriched in pathways Regulation of Insulin-like growth Factor (IGF) transport and uptake by insulin-like Growth Factor Binding Protein (IGFBP), Regulation of secretion, and Protein-lipid complex remodeling (Figure 5A).

**TABLE 2:** The most significantly differently expressed genes in HEL100.2 and other FXS NPCs at each studied time points. *p*-values were adjusted by the method of Benjamini and Hochberg (Benjamini and Hochberg, 1995).

Neurosphere			Day 1			Day 7		
Gene	Log_2_FC	*p*-value	Gene	log_2_FC	*p*-value	Gene	log_2_FC	*p*-value
*DPP10*	6.86	0.000015	*DPP10*	7.09	0.000010	*DPP10*	5.34	0.00011
*glochyby*	−8.00	0.00012	*ZNF736*	−4.00	0.00022	*FIRRE*	3.77	0.00011
*AC134882.1 *	−3.75	0.00017	*FIRRE*	3.13	0.0012	*TACR1*	−7.56	0.00011
*karflerby*	−4.92	0.00030	*DOCK8*	4.04	0.0016	*CTRB1*	3.59	0.00011
*IAH1*	4.82	0.00030	*IAH1*	4.46	0.0023	*B3GAT2*	−4.33	0.00026
*LOC100507412*	−6.96	0.00030	*LRRC4C*	6.88	0.0023	*IAH1*	5.09	0.00048
*AC242006.2*	−4.57	0.00030	*glochyby*	−5.55	0.0025	*SLC6A5*	−5.56	0.00051

*DPP10*, Dopeptidyl Peptidase Like 10; *IAH1*, Isoamyl Acetate Hydrolyzing Esterase 1; *ZNF736*, Zinc Finger Protein 76; *DOCK8*, Dedicator Of Cytokinesis 8; *LRRC4C*, Leucine Repeat Containing 4C; *TACR1*, Tachykinin Receptor 1; *CTRB1*, Chymotrypsinogen B1; *B3GAT2*, Beta-1,3-Glucuronyltransferase 2; Solute Carrier Family 6 Member 5 (*GLYT2*).

### Expression of genes implicated in audiogenic seizures in neural progenitors derived from the fragile X syndrome donor with epilepsy

The absence of FMRP in VGLUT2-expressing neurons located in subcortical brain regions has been shown to be critical to the AGS phenotype of *Fmr1* KO mice (Gonzalez et al., 2019). NPCs express VGLUT2 and its inhibition can promote neuronal differentiation by decreasing excitotoxicity (Sanchez-Mendoza et al., 2017). We found that the expression level of *SLC17A6*, encoding VGLUT2, was lower in NPCs representing FXS + epilepsy phenotype when compared to FXS NPCs derived from donors without epilepsy and control NPCs at all studied time points (Figure 5B), but the result did not reach statistical significance when adjusted for multiple comparisons.

Glutamatergic neurons in the inferior colliculus contribute to the AGS phenotype in *Fmr1* mice (Gonzalez et al., 2019). Axon terminals of VGLUT2-positive excitatory neurons extending from the inferior colliculus to multiple brain regions show high expression levels of *DPP10* (Fujimoto et al., 2021) that, compared to other FXS NPCs, was the most significantly increased transcript in HEL100.2 NPCs throughout the sampling period (Figure 5C). DPP10 interacts with the voltage-gated K+ channel 4 (Kv4) (Ren et al., 2005), whose dysfunction has been associated with epilepsy (Singh et al., 2006) and ASD with concomitant epilepsy (Lin et al., 2018). Kv4.2 is highly expressed in VGLUT2-positive excitatory axon terminals and FMRP has been reported to regulate potassium channel subtypes in a cell type-dependent manner (Lu et al., 2016). DPP10 dysregulation could through control of Kv4.2 function, contribute to auditory hypersensitivity, auditory startle, and the epilepsy phenotype in FXS. Also, expression of *SLC6A5*, encoding the glycine transporter 2 (GLYT2), is located to presynaptic elements of glycinergic neurons in brain stem (Zafra et al., 1995) and its expression was reduced in HEL100.2 NPCs at D7 compared with other FXS NPCs (Figure 5D). *GLYT2* mutations cause hereditary hyperekplexia, which is characterized by an exaggerated tactile or auditory startle response leading to hypertonia and apnea episodes (Carta et al., 2012). Individuals with FXS have severe impairments in sensorimotor gating seen as disrupted prepulse inhibition (PPI) of acoustic startle (Frankland et al., 2004). Although *Fmr1* KO mice show impaired sensorimotor gating and learning, PPI is enhanced (Chen and Toth, 2001), suggesting that compensatory mechanisms with species-specific differences exist. The magnitude of the PPI impairments in FXS children predicts severity of intellectual disability and problems in attention, adaptive behavior, and autistic phenotype (Frankland et al., 2004). Compromised compensatory increase in *GLYT2* expression may associate with epilepsy and potentially with more severe FXS phenotype (Kaufmann et al., 2017).

## Discussion

Manifestation of the phenotype in neurodevelopmental disorders reflects complexity of CNS function that is shaped by genetic and environmental factors. Clinical diagnosing fails to consider the mechanisms underlying differences in symptoms and comorbidities, which can affect treatment responses. Patient-specific stem cells and reprogramming technology provide new opportunities to study molecular backgrounds of developmental neuropsychiatric disorders. Patient-specific iPSCs facilitate the consideration of individual differences while offering a robust alternative to animal models. Human iPSCs may offer potential in screening drugs for efficacy and toxicity. These impacts are particularly considerable in studies of CNS conditions, where research has been limited by non-invasive methodology and paucity of tissue samples. Our study identified FXS-specific gene expression changes in early-stage NPCs by utilizing human iPSCs. We found reduced *LYNX1* expression that *via* tPA-dependent mechanism could affect process growth in FXS NPCs and may in combination with patient-specific transcriptional regulation of genes related to epilepsy contribute to the FXS epilepsy phenotype.

Absence of FMRP resulted in increased expression of non-coding RNAs and reduced RNA complexity during early differentiation of NPCs. These changes were in line with increased expression of *PPIL3*, which is a spliceophilin regulating mRNA splicing (Rajiv and Davis, 2018). In addition, our study showed for the first time that *RAD51C* expression was altered in FXS NPCs, suggesting that *RAD51C* contributes to the increased variability and instability of the tandem repeat sequences observed in human FXS progenitors (Sheridan et al., 2011). RAD51 protein-selected sequences share similarity with DNA segments in the triplet repeat expansion in FXS and depletion of RAD51 is associated with reduced instability of the tandem repeat sequences in FXS (Kononenko et al., 2018). Increased expression of *GUCY1A2* found in FXS NPCs may reduce cAMP signaling *via* the NO/cGMP-PDE2-pathway (Polito et al., 2013), which coincides with increased activity of Pde2a in *Fmr1* KO mouse brain as well as with the observation that targeting Pde2a rescues adult *Fmr1* KO mouse phenotype (Maurin et al., 2019).

FXS NPCs expressed less *LYNX1* than control NPCs, which could critically affect differentiation of FXS progenitors leading to abnormalities in neuronal cell populations and neuronal network formation. *LYNX1* is a member of the Ly6/uPAR/neurotoxin family (Miwa et al., 2019). LYNX1 is implicated in visual attentional deficits observed in the *Fmr1* KO mouse (Falk et al., 2021) and its role in the pathophysiology of FXS is in agreement with its function as a potential target of FMRP (Darnell et al., 2011). However, its developmental expression change has not been shown in previous cortical transcriptome or proteomics analyses on *Fmr1* KO mice (Xu et al., 2018; Das Sharma et al., 2019). *LYNX1* expression was temporally reduced in pluripotent FXS NPCs and the reduction was no longer visible at day 30 in ESC-derived NPCs (Peteri et al., 2021), suggesting that LYNX1 is involved in altered control of molecular signaling that regulates fate of FXS NPCs. An increase in LYNX1 expression as a cholinergic brake occurs normally at the end of critical period for visual plasticity (Morishita et al., 2010). *Lynx1* KO mice, *Fmr1* KO mice and human FXS patients display similarly increased pools of immature dendritic spines (Comery et al., 1997; Irwin et al., 2001; Falk et al., 2021).

LYNX1 is a nicotine modulator and has a negative allosteric action on nAChR function (Miwa, 2021). The neuronal nAChRs are assembled from *a* and *ß* subunits, and the subunit composition of the pentameric receptors influences receptor properties (Nichols et al., 2014). The absence of LYNX1 enhances sensitivity to nicotine and affects excitatory/inhibitory balance (Morishita et al., 2010). Nicotinic acetylcholinergic transmission regulates interneuron function and LYNX1 serves as a regulator of the convergence of GABAergic and nicotinic systems in a specific subpopulation of interneurons. Cortical parvalbumin (PV) interneurons are a specific site of LYNX1 expression (Demars and Morishita, 2014). Embryonic deletion of *Fmr1* in cortical excitatory neurons during the developmental window of PV cell maturation reduces PV expression and activity of PV cells, leading to behavioral deficits and impaired auditory cortical responses in *Fmr1* KO mice (Wen et al., 2018). Impaired interneuron function is considered an important ictogenic factor and loss of interneurons is documented in chronic epilepsies (Dudek, 2020). Our study suggested that *LYNX1* contributes to hypofunction of PV interneurons implicated in cortical hyperexcitability in FXS and likely more broadly in ASD (Filice et al., 2020).

*Lynx1* KO increases tPA activity and the immature spine morphology in *Lynx1* KO mice is corrected in the absence of tPA (Bukhari et al., 2015). Here, we showed that blocking tPA function with an antibody reduced abnormal process growth in human iPSC-derived FXS NPCs with reduced *LYNX1* expression. Our previous studies showed increased tPA expression in the brain of *Fmr1* KO mice and in mouse NPCs lacking FMRP (Achuta et al., 2014). Furthermore, blocking tPA function prevented enhanced intracellular Ca^2+^ responses to membrane depolarization and corrected a migration defect of doublecortin-immunoreactive cells in differentiating mouse neurospheres (Achuta et al., 2014). Proliferation of FXS NPCs is increased and fate determination of progenitors lacking FMRP is affected leading to an increase in intermediate progenitors in the *FMR1* KO mouse brain (Tervonen et al., 2009; Castrén, 2016; Utami et al., 2020). These changes likely contributed to the differences seen in the process growth of FXS vs. control progenitors. There is evidence that BDNF-mediated mechanisms are involved in augmented functional responses and morphological changes in NPCs lacking FMRP (Louhivuori et al., 2009; Achuta et al., 2018; Danesi et al., 2018). A genetic deletion of one copy of the *Bdnf* gene, causing 50% reduction of BDNF expression levels, rescued the process growth of *Fmr1* KO mouse neural progenitors (Uutela et al., 2012). Reduced BDNF was also shown to correct sensorimotor deficits in *Fmr1* KO mice, indicating correlation between defective process growth and neuronal circuit function caused by FMRP deficiency (Uutela et al., 2012). The plasminogenic activity of tPA is important for processing precursor form of BDNF (proBDNF) to active mature BDNF (Pang et al., 2004) and dysregulated tPA expression may play a critical role *via* BDNF-mediated mechanisms in FXS neurogenesis. In addition, plasmin-independent enzymatic activity of tPA can potentiate glutamatergic signaling and contribute to augmented intracellular Ca^2+^ responses to activation of glutamate receptors in FXS NPCs (Achuta et al., 2017; Danesi et al., 2018).

Although glutamatergic responses were similarly increased in all FXS NPCs compared to controls, we observed difference in the gene expression profile between NPCs derived from a FXS donor with epilepsy and donors without epilepsy. Around 20% of FXS individuals suffer from epilepsy, typically childhood epilepsy with centrotemporal spikes within diverse seizure types (Berry-Kravis, 2002). The donor of HEL100 NPCs displayed epilepsy with centrotemporal spikes typical to FXS. Several genes were differentially expressed in the transcriptome of NPCs derived from the HEL100.2 FXS iPSC line when compared with the gene expression in NPCs derived from FXS donors without epilepsy. Analysis of differently expressed genes in cholinergic synaptic pathway showed high variation in CHRNB2 expression and a tendency toward higher expression levels in FXS NPCs without epilepsy than in control NPCs and FXS NPCs with epilepsy. Since mutations of the *CHRNB2* gene are associated with autosomal dominant nocturnal frontal lobe epilepsy (Diaz-Otero et al., 2008), our data suggest that *CHRNB2* may be involved in mechanisms leading to an increased risk of epilepsy in FXS. However, due to the complexity of the cholinergic regulation, despite clear genetic association with epilepsy the role of *CHRNB2* in the pathogenesis of epilepsy is still unclear (Becchetti et al., 2015).

We showed that FXS NPCs during early differentiation *in vitro* display specific gene expression changes, which are consistent with the impact of lack of FMRP seen on molecular and functional properties of neuronal cells and clinical phenotype (Davis and Broadie, 2017; Salcedo-Arellano et al., 2020; Utami et al., 2020). Observed gene expression abnormalities in NPCs were also in agreement with currently recognized potential treatment targets for FXS. As a new discovery, we found reduced *LYNX1* expression in FXS NPCs, indicating involvement of altered cholinergic system in developmental defects in FXS. LYNX1-mediated mechanisms could be potentially linked to the increased risk of epilepsy based on a patient-specific gene expression profile enriched with known genes associated with epilepsy in NPCs derived from one FXS donor with epilepsy. Many of the dysregulated genes were also associated with ASD and more severe clinical forms of FXS consistent with often overlapping epilepsy and ASD in FXS (Kaufmann et al., 2017). Furthermore, epilepsy phenotype-related genes were enriched in Reactome pathway of regulation of IGF transport and uptake by IGFBP in agreement with previous studies showing that increased IGF signaling exacerbates the AGS phenotype in the *Fmr1* KO mice (Wise, 2017). IGF pathway has been recognized as a potential therapeutic target in FXS (Vahdatpour et al., 2016; Wise, 2017) and our studies support the hypothesis based on studies of mice, but also urge attention to possible individual variation in drug responses. Patient-specific variation in NPCs may reflect individual developmental differences in NPC fate determination, differentiation, and migration, affecting neuronal network formation and determining manifestation of the phenotype. Since the lissencephalic mouse brain differs substantially from the gyrated human brain, human iPSC models are valuable in defining human-specific responses in preclinical studies. In clinical settings, individual differences in treatment responses complicate the clinical work. Human iPSC research offers human cell-based models to tackle this issue. The present study is limited to a small number of FXS iPSC lines and additional larger studies are needed to confirm the data and further explore FXS phenotype-related changes in NPCs.

## Data Availability

The data supporting the conclusions of this study are available from the corresponding author upon reasonable request. The human microarray data reported and used in this paper are available at the Gene Expression Omnibus database under accession no. GSE1033965 and GSE216875.
