# Supplementary material for: Reduced LYNX1 expression in transcriptome of human iPSC-derived neural progenitors modeling fragile X syndrome
Source: Front Cell Dev Biol. 2022 Nov 21;10:1034679. doi: 10.3389/fcell.2022.1034679 (PMC9731341; doi:10.3389/fcell.2022.1034679)
Supplement: Supplementary file 3 [file Table1.pdf]

Supplementary Material

Supplementary Table. Genes that were differently expressed (adjusted $p < 0.05$) in NPCs derived from HEL100 and other FXS hiPSC lines at all studied time points (NS, D1, and D7).

	Neurosphere		Day 1		Day 7	
Gene	log2FC	p-value	log2FC	p-value	log2FC	p-value
DPP10	6.858778	1.5142E-05	7.087068	1.014E-05	5.3393555	0.00010966
ZNF736	-2.5309285	0.00109825	-3.9987905	0.00021808	-2.16857	0.01128881
FIRRE	2.3830215	0.00105857	3.12616	0.00117678	3.7714655	0.00010966
IAH1	4.8160555	0.00029959	4.459336	0.0023425	5.0883695	0.00047622
LRRC4C	6.658221	0.00052744	6.8815335	0.0023425	4.0100985	0.0320567
TACR1	-3.7673035	0.00504203	-5.0994655	0.00402182	-7.562362	0.00010966
C11orf74	5.708917	0.00106215	6.0139125	0.00416627	5.578516	0.00480821
SPINK1	7.9326135	0.00303234	9.613624	0.00430627	10.75985	0.00149336
LYPD1	-3.192889	0.00037789	-2.765601	0.00430627	-1.95552	0.02350267
ARL17B	-2.905166	0.00105857	-2.882942	0.00510522	-2.829404	0.00478651
B3GAT2	-2.4686315	0.00394357	-2.985124	0.0052815	-4.3259235	0.00026312
C1orf61	-3.556077	0.0121023	-5.664433	0.0052815	-6.979525	0.00107229
SI	2.436426	0.01833718	3.602563	0.00675855	2.7092385	0.02926239
DCT	-6.2827505	0.00887808	-7.930225	0.00833758	-5.848379	0.03705418
BCAN	-2.101919	0.01975599	-2.9911825	0.01045531	-2.7070815	0.01693818
PCDH19	-4.734726	0.00087738	-3.681858	0.01549008	-2.99952	0.03494451
FYN	-2.86593	0.00078219	-2.18559	0.01598312	-2.560595	0.00510933
REC8	3.105359	0.00073931	2.3085645	0.01643149	2.3552325	0.01276548
POU5F1	5.289889	0.0287178	7.5541225	0.01730337	5.971276	0.04549801
TM4SF4	3.7408725	0.004136	3.6702635	0.01798406	4.9918015	0.00210797
PARM1	4.825601	0.00578249	5.0003875	0.01803155	4.211612	0.03550672
MIR302C	5.7757035	0.01419271	6.840728	0.01924049	7.03466	0.01635103
H19_3	2.9774615	0.0017529	2.451418	0.02005684	3.549237	0.0017568
AGT	6.0331685	0.00525103	5.9042835	0.02005684	7.6247295	0.00478651
TDGF1	5.425209	0.04312588	7.863876	0.02241643	7.231729	0.03174182
ADAM22	-2.375143	0.00434304	-1.984743	0.04097873	-1.876295	0.04556015
PAQR5	2.1621435	0.00412561	1.7361245	0.04873416	2.0707805	0.01823973
AFP	9.266314	0.01661087	9.462214	0.04873416	13.910024	0.00510933
MUC13	4.3919505	0.00541011	3.680165	0.04943309	3.8592365	0.0320567
A2M	4.504662	0.0360579	5.4528275	0.04943309	6.160204	0.02350267
